# The Effect of Non-Surgical Periodontal Therapy on Pentraxin-3 Concentration in Gingival Crevicular Fluid of Chronic Periodontitis Patients: A Clinical Trial

**DOI:** 10.30476/DENTJODS.2021.90647.1510

**Published:** 2022-09

**Authors:** Asieh Mozaffari, Mahtab Samieifar, Bahare Abd Nikfarjam, Monirsadat Mirzadeh, Shadi Assareh, Samira Mohammad Mirzapour

**Affiliations:** 1 Dept. of Periodontology, Dental Caries Prevention Research Center, Qazvin University of Medical Sciences, Qazvin, Iran; 2 Dept. of Immunology, School of Medicine, Qazvin University of Medical Sciences, Qazvin, Iran; 3 Metabolic Disease Research Center, Research Institute for Prevention of Non-Communicable Diseases, Qazvin University of Medical Sciences, Qazvin, Iran; 4 Periodontist, Private Practice, Tehran, Ira; 5 Postgraduate Student, Dept. of Periodontics, Student Research Committee, Qazvin University of Medical Sciences, Qazvin, Iran

**Keywords:** Chronic periodontitis, Gingival crevicular fluid, Pentraxin 3

## Abstract

**Statement of the Problem:**

Periodontitis is a multifactorial disease caused by periopathogens and its severity is determined by the host immune response. Gingival crevicular fluid (GCF) can be used for non-invasive testing to assess the host response in periodontal treatment. Pentraxins are the classic mediators of inflammation and pentraxin-3 can be used as a marker to assess response to therapy, which was investigated in this study.

**Purpose:**

This study aimed to assess the effect of non-surgical periodontal therapy on GCF level of pentraxin-3 in patients with chronic periodontitis.

**Materials and Method:**

25 patients with chronic periodontitis (CP) and 25 periodontally healthy controls were evaluated. Pocket probing depth, clinical attachment loss, plaque index, gingival index, and bleeding on probing were measured in both groups. GCF samples were collected using paper strips to assess the level of pentraxin-3. In the CP group, GCF samples were collected from the highest clinical attachment loss, pocket probing depth, and bone loss at baseline and six weeks after non-surgical therapy. The level of pentraxin-3 in the GCF was quantified by enzyme-linked immunosorbent assay (ELISA). Data were analyzed using SPSS version 23.

**Results:**

Pentraxin-3 in GCF of CP patients before treatment (6.72±4.63 ng/mL) was higher than the control group (4.43±2.85 ng/mL). Pentraxin-3 in patients after non-surgical therapy (3.2±2.66 ng/mL) decreased significantly compared to the baseline (*p*= 0.04) and its level after treatment was not significantly different from the control group (*p*= 0.14).

**Conclusion:**

Pentraxin-3 in GCF of CP patients was higher than healthy controls and decreased in response to non-surgical periodontal therapy. Thus, it can be used as an inflammatory marker for detection of patients at risk of CP. However, further studies with larger samples and longer follow-ups in different populations are required to confirm our findings

## Introduction

Periodontitis is a chronic inflammatory disease that is caused by polymicrobial infection [ 1 ]. It results from the interaction of periodontal pathogens and host inflammatory responses [ 2 ]. Chronic periodontitis (CP) is often diagnosed based on radiographic findings and clinical parameters, including pocket probing depth (PD) and clinical attachment loss (CAL). However, measurement of these parameters does not provide any information about the current status of disease or its future course [ 3 ]. Biomarkers are capable of providing additional information on standard clinical indices [ 4 - 5 ].

The progression of periodontal disease is site-specific and an increase in the count of lymphocytes, neutrophils, and macrophages is seen in primary lesions [ 6 ]. The gingival crevicular fluid (GCF) also increases during gingival inflammation [ 5 , 7 ]. Periodontal inflammation is regulated by a combination of chemokines and cytokines [ 8 During the primary phases of CP, bacteria invade the connective tissue [ 9 ]. In response to bacterial endotoxins, acute phase proteins are produced that activate an inflammatory cascade [ 10 - 11 ]. Inflammatory reactions increase the level of acute phase proteins in GCF [ 12 ].

Pentraxins can be categorized into two groups; the first group is short pentraxins, and the second is long pentraxins [ 13 ]. The first group includes C-reactive protein and serum amyloid P component. They are acute phase proteins produced in response to inflammatory mediators in the liver [ 14 ]. The first long pentraxin is Pentraxin-3 [ 8 ], which plays a vital role in the inherent resistance to pathogens, regulation of inflammatory reactions, and removal of apoptotic cells [ 9 ]. Pentraxin-3 is produced in response to pro-inflammatory signals such as bacterial constituents (lipopolysaccharides, lipoarabinomannans, and outer membrane proteins), Toll-like receptor engagement, and cytokines (tumor necrosis factor-alpha and Interleukin1β) [ 15 ]. It is produced and released by diverse cell types, primarily by cells frequently found in the periodontal tissue, including fibroblasts, neutrophils, dendritic cells, epithelial cells, macrophages, and endothelial cells [ 4 , 16 ].

One of the main challenges in periodontology is finding a reliable high-sensitivity and -specificity molecular marker for early detection of periodontal tissue destruction. In the recent years, a number of cellular mediators and enzymes have been identified in the GCF with predictive value in detection of tissue loss in CP [ 4 , 17 - 18 ]. Due to extra-hepatic synthesis of pentraxin-3 in the inflamed tissues (in contrast to C reactive protein), it is believed that the level of pentraxin-3 is an independent indicator of disease activity [ 19 ]. In addition, pentraxin-3 can activate the complement system, which highlights its role in regulation of inflammation and innate immune responses [ 20 ]. A review study done by Katharia *et al.* [ 8 ] showed that pentraxin-3 might be used as biomarker in the diagnosis of periodontal disease; besides, it might have clinical implication in diagnosing the “at site” inflammatory status of periodontal disease.

Since there is paucity of research on the effects of non-surgical periodontal therapy (scaling and root planing) on GCF level of pentraxin-3, this study aimed to assess the effect of non-surgical periodontal therapy on GCF level of pentraxin-3 in patients with chronic periodontitis [ 21 - 22 ].

## Materials and Method

The study was approved in the Ethics Committee of Qazvin University of Medical Sciences (IR.QUMS. REC.1396.188) and registered in Iranian Registry of Clinical Trials (code: IRCT20190717044253N). 

This clinical trial evaluated 25 patients with CP and 25 individuals with healthy periodontium as controls. Research sample were 25 in each group, given alpha=0.5 and 1-beta=0.8. Sampling was done in a sequential manner until the sample size was reached. 

Selection of CP patients was based on the criteria set in the International Workshop of Periodontal Disease in 1999 [ 23 ]. The controls were selected among healthy individuals between 25 to 50 years old who had no history of periodontal disease and had bleeding on probing (BOP) less than 20%. In addition, they did not have any site with PD>3 mm or CAL and had no radiographic indication of bone loss on their full-mouth radiographs taken for their treatment. 

The patient group comprised of 25 patients with generalized moderate CP between 25 to 50 years old. They had sites with PD≥5 mm and CAL≥3 mm compatible with the amount of accumulated plaque and had radiographic indication of bone loss.

The exclusion criteria were systemic conditions, including respiratory and cardiovascular diseases, diabetes mellitus, human immunodeficiency syndrome, chemotherapy, pregnancy or nursing, antibiotic therapy, medication intake, periodontal treatment in the past 6 months, smoking, infectious diseases and any sign/ symptom of infection. All participants were informed of the study protocol, and a written informed consent was obtained.

Full-mouth clinical periodontal examination (except for third molars) was carried out for both the patient and control groups including the assessment of PD and CAL at 6 sites around each tooth using a Williams periodontal probe (Hu-Friedy, Chicago, IL). The plaque index (PI) [ 24 ], bleeding index (BI) [ 25 ] and gingival index (GI) [ 26 ] were recorded. In order to avoid GCF contamination with blood resulting from the probing of inflamed sites, samples were collected on the subsequent day.

Participants then received oral hygiene instructions (the rolling method twice a day). Phase one periodontal therapy was performed for CP patients in two sessions with one-week interval, and included scaling and root planing with hand and ultrasonic instruments. No antibiotic or anti-inflammatory agent was prescribed for the patients. CP patients were recalled six weeks after the second treatment session. GCF was collected again from the same areas and clinical parameters (CAL, PD, PI, BI, and GI) were measured again. All procedures were performed by the same operator (a post-graduate student of periodontology). The patients were recalled two weeks after the second scaling session and their adherence to oral hygiene instructions was evaluated. Supragingival prophylaxis was also performed for them.

### Collection of GCF

Periopaper collection strips (Oraflow Inc., Smithtown, NY, USA) were employed for GCF collection. In CP patients, GCF was collected from the region with the highest CAL, inflammation, and radiographic bone loss. In healthy controls, GCF samples were collected from non-inflammatory areas with PD<3 mm without BOP. To prevent saliva contamination, isolation was performed with cotton rolls. A gauze was employed for the removal of supragingival plaque and the teeth were dried with air spray. Strips were inserted in to the crevice until mild resistance was felt and remained there for one minute. Samples with detectable blood contamination were excluded. Then, collected paper strips were placed in micro-centrifuge tubes and stored at -80°C until laboratory analysis. All samples were coded and sent blindly to the laboratory. Samples were collected at baseline before scaling and root planing and also at 6 weeks after the completion of phase I periodontal therapy. 

### Assay procedure

The level of pentraxin-3 in GCF samples was quantified by enzyme -linked immunosorbent assay (ELISA) technique using human pentraxin-3/TSG-14 immunoassay (Catalogue no. DPTC30; R&amp;D SYSTEMS, Minneapolis, MN). The method of measurement was quantitative sandwich enzyme immunoassay. Manufacturer’s instructions were followed for each assay. Pentraxin-3 biotinylated antibody was added to the wells and incubated. Samples and pretreated standards were added to the wells after washing the plates. The wells were then emptied and rinsed with wash buffer four times. Then, human pentraxin-3 conjugate was added to each well. After washing, each well was added a substrate solution and incubated at room temperature for 30 minutes away from light. Next, stop solution was added to each well. There was a color change in the solution from blue to yellow. ELISA reader (Biotek–EIX 800, Biotek Vermont, USA) was employed to measure the optical density of solutions in wells within 30 minutes at 450 nm wavelength. Using the standard curve of optical density, the concentration of pentraxin-3 was quantified. The concentration results were measured in ng/ml. All laboratory tests were performed in the Immunology Department of Qazvin University of Medical Sciences.

### Statistical analysis

Data were analyzed using SPSS 23 (SPSS Inc., IL, USA). Kolmogorov-Smirnov test was used for testing the normality of data distribution. The two groups were compared using a Student t-test, and paired t-test was used for comparisons within the groups. The Pearson’s correlation test was applied to assess the correlation between the clinical parameters and concentration of pentraxin-3 separately in the control and patient groups before and after the treatment. P ≤0.05 was considered to be statistically significant. The power of the study was determined as 80% and α=0/05.

## Results

A total of 25 CP patients and 25 healthy controls were evaluated. There were 12 females (48%) and 13 males (52%) in the CP group and 13 females (52%) and 12 males (48%) in the control group. There were no significant differences between the two groups with respect to gender (*p*= 0.61). The mean age was 38.44±8.06 years in the CP group and 34.52±6.24 years in the control group and the two groups were not significantly different in this respect (*p*= 0.06). The mean pentraxin-3 concentration was 6.74±4.63 ng/mL in the CP group and 4.43±2.85 ng/mL in the control group. The two groups were significantly different in the concentration of pent-raxin-3 at baseline (student t-test, *p*= 0.04). All clinical parameters in the CP group were significantly higher than those of the healthy controls at baseline *p*< 0.001, Table 1). (Student t-test, all CP patients had BOP while the control subjects did not have BOP. The mean concentration of pentraxin-3 was 3.2±2.66 ng/mL in the CP group after the treatment (CP2 group). After periodontal therapy, there was no significant difference between patients and controls in concentration of pentraxin-3 (Student t-test, *p*= 0.14). In the CP group, all clinical parameters were significantly higher than those of the control group after treatment (Student t-test, *p*<0.001) except for PI (Student t-test, *p*=0.08). The sampling areas did not have BOP in the control group and in the CP group after treatment. The concentration of pentraxin-3 significantly decreased after periodontal therapy in the CP group (6.72±4.83 ng/ mL before and 3.2±2.66 ng/mL after treatment, paired t-test, *p*= 0.001). All clinical parameters in the CP group, before treatment were significantly higher than those after treatment (paired t-test, *p*=0.001, Table 1). The results of Pearson’s correlation test revealed a significant correlation between the level of pentraxin-3 and full-mouth PD (*p*= 0.001, r= 0.6) and site PD (*p*= 0.01, r=0.5) in the CP group before treatment (Table 2). Significant correlations were noted between the concentration of pentraxin-3 and full-mouth GI (*p*=0.02, r=0.15) and full-mouth PI (*p*= 0.01, r=0.54) in the CP group after treatment (Table 2). In the control group, there was no significant correlation between the level of pentraxin-3 and any of the clinical parameters (*p*> 0.05) (Tables 2^3^ to 4).

**Table 1:** Periodontal clinical parameters in chronic periodontitis(CP) and control groups

	BI(%)	SCAL (mm)	TCAL (mm)	FMCAL (mm)	SPD (mm)	TPD (mm)	FMPD (mm)	SGI	TGI	FMGI	SPI	TPI	FMPI
CP group	72.77±29.28	5.72±1.3	3.87±1.29	2.98±1.06	5.8±0.91	3.55±0.78	2.88±0.71	2.76±0.43	2.6±0.49	2.35±0.46	2.6±0.64	2.42±0.69	1.99±0.69
CP2 group	19.8±11.7	3.05±1.23	2.46±1.25	1.96±0.91	2.65±1.18	1.95±0.75	1.82±0.74	0.4±0.5	0.56±0.48	0.57±0.48	0.68±0.15	0.460.44	0.43±0.31
Control group	6.04±3.66	0	0	0	1.84±0.37	1.44±0.42	1.39±0.32	0	0	0.17±0.15	0.28±0.45	0.21±0.31	0.3±0.13

FMPI= Full Mouth Plaque Index; TPI= Tooth Plaque Index; SPI= Site Plaque Index; FMGI= Full Mouth Gingival Index; TGI=Tooth Gingival Index; SGI= Site Gingival Index; FMPD= Full Mouth Probing Depth; TPD= Tooth PD; SPD= Site PD; FMCAL= Full Mouth Clinical Attachment Loss; TCAL= Tooth Clinical Attach-ment Loss; SCAL= Site Clinical Attachment Loss; BI= Bleeding Index.

**Table 2:** Pearson correlations for Pentraxin-3 values

Variables	CP group	CP2 group	Control group
Full mouth PI			
Correlation	-0.05	0.54	-0.13
*p*Value	0.79	0.01	0.51
Full mouth GI			
Correlation	-0.01	0.51	0.25
*p*Value	0.94	0.02	0.21
Full mouth PD			
Correlation	0.6	0.07	-0.01
*p*Value	0.001	0.74	0.95
Full mouth CAL			
Correlation	0.19	-0.16	NA
*p*Value	0.34	0.49	NA
Tooth PI			
Correlation	-0.04	0.18	-0.11
*p*Value	0.82	0.42	0.57
Tooth GI			
Correlation	-0.009	-0.07	NA
*p*Value	0.96	0.76	NA
Tooth PD			
Correlation	0.27	-0.02	-0.049
*p*Value	0.18	0.09	0.81
Tooth CAL			
Correlation	-0.028	-0.09	NA
*p*Value	0.89	0.7	NA
Site PI			
Correlation	-0.22	0.07	-0.16
*p*Value	0.27	0.74	0.44
Site GI			
Correlation	-0.14	0.22	NA
*p*Value	0.49	0.34	NA
Site PD			
Correlation	0.5	-0.26	0.31
*p*Value	0.01	0.26	0.12
Site CAL			
Correlation	0.19	0.01	NA
*p*Value	0.35	0.93	NA

The level of significance was set at 0.05. The *P* values that were less than 0.05 were significant and are in bold

NA=Not Applicable

PI= Plaque Index; GI=Gingival Index; PD= Probing Depth; CAL= Clinical Attachment Loss

**Table 3:** Pearson correlations of Pentraxin-3 values for bleeding index

Variables	CP group	CP2 group	Control group
Bleeding Index			
Correlation	0.15	0.44	-0.16
*p*Value	0.44	0.052	0.42

The level of significance was set at 0.05. The *p* Values that were less than 0.05 were significant and are in bold. NA=Not Applicable

CP=Chronic periodontitis

**Table 4:** Pentraxin-3 concentration (ng/ml) of study groups

Group	n	Mean ± SD
CP group	25	6.74 ± 4.63 ǁ ǂ
CP2 group	20	3.2 ± 2.66
Control	25	4.43 ± 2.85

ǁ *p* < 0.05, significantly higher than control group.

‡ *p* < 0.05, significantly higher than CP2 group.

CP=Chronic periodontitis

## Discussion

This study aimed to assess the concentration of pentraxin-3 in CP patients before and after non-surgical periodontal therapy compared to the control group. The number of males and females was the same in the CP and control groups. Also, the participants were in the range of 25 to 50 years. It minimized the effect of gender and age on the results. The current findings confirmed the presence of pentraxin-3 in GCF of both CP patients and healthy controls. The concentration of pentraxin-3 in the CP group before treatment was significantly higher than that of the control group. Periodontal therapy in CP group significantly decreased all periodontal parameters, which confirmed the efficacy of non-surgical periodontal therapy. Since PD decreased and there was no BOP after periodontal therapy, the patients did not need to undergo periodontal surgery. The concentration of pentraxin-3 significantly decreased following periodontal therapy. No significant difference was noted in the concentration of pentraxin-3 between the CP group after treatment and the control group. Also, all clinical periodontal parameters of CP group after treatment were higher than the control group, except for PI. This can indicate faster reduction of pentraxin-3 concentration due to resolution of inflammation compared to clinical parameters or may be due to the time of sampling and assessment after treatment. 

The concentration of pentraxin-3 in patients before treatment had a significant correlation with full-mouth PD and site PD. After treatment, there was a significant correlation between pentraxin-3 concentration and oral GI and PI. The high standard deviation of the pentraxin-3 level before and after treatment in the CP group and also in the control group can be due to the small sample size in the two groups. The findings are consistent with Pradeep *et al.* [ 19 ]. They reported not only a significant increase in the concentration of pentraxin-3 in the plasma and GCF of patients with gingivitis compared to healthy controls but also in patients with periodontitis compared to those with gingivitis. Minimum values were recorded in the healthy group.

In a study by Elmonem *et al.* [ 27 ], the mean concentration of pentraxin-3 in GCF of CP patients was significantly higher than patients with gingivitis and healthy controls. Also, the concentration of pentraxin-3 was significantly correlated with clinical parameters. Fujita *et al.* [ 6 ] showed a strong correlation between the concentration of pentraxin-3 in GCF and site clinical parameters. In current study, only the site PD had a significant correlation with the concentration of pentraxin-3 in the GCF, which may be due to different sample size, inclusion and exclusion criteria, and study design, as Fujita *et al.* [ 6 ] followed a split-mouth design.

To the best of authors’ knowledge, only two previous studies performed by Afifi *et al.* [ 22 ] and Mohan *et al.* [ 21 ], evaluated the level of pentraxin-3 in GCF of patients with CP before and after non-surgical periodontal therapy. In the study by Afifi *et al.* [ 22 ], GCF samples were collected at baseline and 8 weeks after treatment completion. They showed significantly higher concentration of pentraxin-3 in CP group compared to the control group at baseline. The concentration of pentraxin-3 in the CP group significantly decreased after treatment compared to baseline, but it was still higher than the control group.

In the present study, the mean concentration of pentraxin-3 after treatment was not significantly different in CP and control groups. Afifi *et al.* [ 22 ] only evaluated female patients and collected samples at 8 weeks while we evaluated both males and females and collected samples at 6 weeks after completion of treatment. Also, patients were recalled after 2 weeks and received supra-gingival prophylaxis. These factors may explain the difference in the results of the two studies. 

In our study, the GI decreased from 68% at baseline to 19.8% after treatment. This reduction was significantly greater than that reported by Afifi *et al.* [ 22 ], since in their study, GI decreased from 66% to 31%. This indicates greater resolution of inflammation in our study and subsequently greater reduction in the concentration of pentraxin-3 in GCF and absence of a significant difference in its concentration between the CP and control groups. 

A study by Mohan *et al.* [ 21 ] investigated the effect of nonsurgical periodontal therapy on pentraxin 3 levels in smokers and nonsmokers with CP. In this study, Pentraxin -3 levels at baseline in patients with CP (smokers and non-smokers) were found to be significantly higher than both groups of clinically healthy subjects (smokers and non-smokers). They showed that scaling and root planing improves clinical parameters and significantly reduces Pentraxin-3 levels (*p*< 0.05) in both chronic periodontitis groups at 2-week follow-up, but these changes were more significant in smokers compared to nonsmokers. 

The current study, consistent with Mohan *et al.* [ 21 ], only assessed non-smokers; hence, pentraxin-3 concentration in the CP group was higher than in the pre-treatment control group, while it significantly decreased after the periodontal therapy. Periodontitis is often diagnosed based on radiographic findings and clinical measurements, which is cost-effective but does not provide any information regarding the current status of disease and prediction of its course in the future. Thus, a main challenge in periodontology is to find a reliable molecular marker for periodontal tissue destruction with high sensitivity, specificity, and applicability [ 28 ].

Considering all the above, it appears that the concentration of pentraxin-3 in GCF may be used to assess the disease status and response to periodontal therapy. However, future studies are suggested to be conducted with larger sample sizes and longer follow-up periods in different populations to increase the reliability and generalizability of our findings. Also, the concentration of pentraxin-3 should be evaluated in GCF of CP patients before and after periodontal surgery. Assessment of the level of pentraxin-3 in the saliva of CP patients before and after surgical and non-surgical periodontal therapy can also be an interesting topic for future research in this field. 

## Conclusion

A higher concentration of pentraxin-3 in GCF of CP patients than in healthy controls may highlight the role of this marker in the pathogenesis of periodontitis. Therefore, it can be used as an inflammatory marker in periodontal disease and can be employed to detect individuals at risk for CP. Nevertheless, Pentraxin-3 concentrations varied considerably in different groups. Therefore, to consider Pentraxin-3 as an inflammatory biomarker of periodontitis in GCF, further studies with larger sample sizes and more extended follow-up periods in different populations are required.

## Conflict of Interests

 The authors declare that they have no conflict of interest.
